# Retromode Scanning Laser Ophthalmoscopy for Choroidal Nevi: A Preliminary Study

**DOI:** 10.3390/life13061253

**Published:** 2023-05-25

**Authors:** Claudia Azzolini, Maura Di Nicola, Francesco Pozzo Giuffrida, Francesca Cappelli, Claudia Bellina, Francesco Viola, Paolo Chelazzi

**Affiliations:** 1 Department of Ophthalmology, Istituto Clinico Città Studi, 20131 Milan, Italy; claudia.azzolini@ic-cittastudi.it (C.A.);; 2 Department of Ophthalmology, Bascom Palmer Eye Institute, University of Miami Miller School of Medicine, Miami 33136, FL, USA; 3 Department of Clinical Sciences and Community Health, University of Milan, 20122 Milan, Italy; 4 Ophthalmological Unit, Fondazione IRCCS Cà Granda Ospedale Maggiore Policlinico, 20122 Milan, Italy

**Keywords:** choroidal nevus, retromode, multimodal imaging, scanning laser ophthalmoscopy

## Abstract

The purpose of the present study was to document pathological findings on retromode imaging in choroidal nevi and evaluate its diagnostic validity, using the confocal scanning laser ophthalmoscope Nidek Mirante (cSLO). A total of 41 choroidal nevi from 41 patients were included. All patients underwent multicolor fundus (mCF), infrared reflectance (IR), green fundus autofluorescence (FAF), dark-field (DF) and retromode (RM) imaging and optical coherence tomography (OCT) scans. We investigated retromode images to evaluate choroidal nevus features by comparing the results with those of mCF, IR, FAF, DF and OCT. In 100% of available images, retromode scanning laser ophthalmoscopy was able to detect choroidal nevi with a characteristic “hypo-retro-reflective” pattern, even the cases not visible on mCF, IR and FAF images. It also made it possible to delineate the margins of lesions with the highest rate of sharpness and accuracy among the imaging modalities examined. These findings seem to demonstrate how RM-SLO is an innovative diagnostic tool to detect and follow up choroidal nevi in a fast, reliable and non-invasive way.

## 1. Introduction

Choroidal nevus is the most common intraocular tumor, with an estimated prevalence of 7% among the white population, often discovered incidentally by ophthalmic examination [1,2]. It commonly appears as a choroidal brown or tan mass with a round or oblong configuration. Although choroidal nevi are histologically benign, they can lead to vision loss and can occasionally evolve into life-threatening malignant melanoma [3]. In recent years, multimodal imaging has become an indispensable tool in ocular oncology, allowing for better definition of tumor features and risk factors for transformation of choroidal nevus into melanoma [4,5,6].

**Figure 1:**
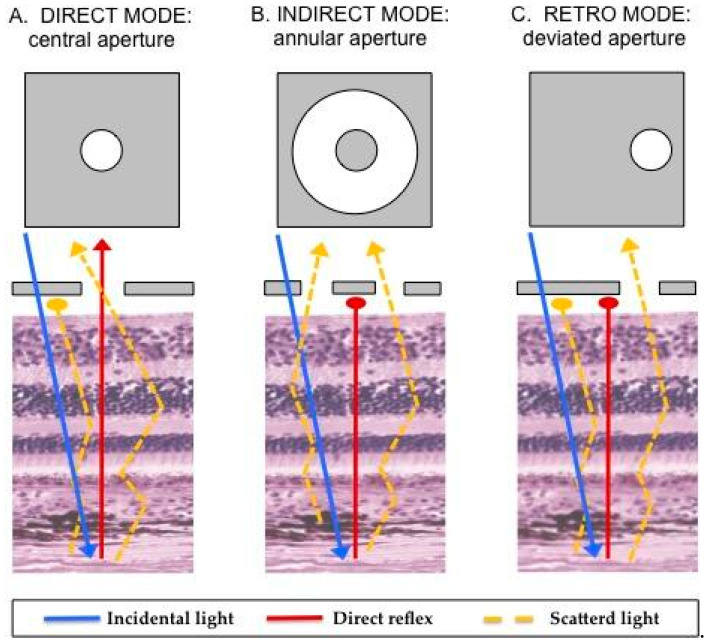
Illustration of the mechanism underlying retromode scanning laser ophthalmoscopy [8,9,10,11]. Once the fundus has been illuminated (incidental light), there are two types of light returning back to the instrument detector: a direct reflex and scattered light. Varying the imaging aperture allows the scanning laser ophthalmoscope to assess the light returning from various parts of the eye. (**A**) Direct confocal mode: a central confocal aperture limits the passage to almost exclusively directly reflected light from the illuminated point on the retina, while other sources of light scatter are blocked. This increases image resolution and contrast. (**B**) Indirect mode, the so-called “dark-field” mode (RA aperture): a central circular stop blocks the directly reflected light, while more widely scattered light can pass through an annular aperture. This creates low-contrast transillumination images. (**C**) Retromode (DR or DL aperture): the opening of the ring aperture is restricted and deviates laterally from the confocal light path. The laterally deviated aperture is used to collect the backscattered light from just one direction, and block the directly reflected light and the light scattered from the other directions. This smaller aperture produces higher-contrast transillumination images with a narrower depth of focus compared to the dark-field images.

Retromode scanning laser ophthalmoscopy (RM-SLO) is a novel noninvasive imaging method provided by the newly introduced scanning laser ophthalmoscope Nidek Mirante (SLO, Nidek Co., Ltd., Gamagori, Japan). Using infrared light (790 nm) that allows for penetration through the deeper layers of the retina and retinal pigment epithelium (RPE) [7,8], RM-SLO provides comprehensive topographic information regarding several deep retinal pathologies, RPE changes and even choroidal lesions [9]. In this imaging modality, the small confocal aperture centered in front of the SLO photodetector is replaced with a laterally displaced aperture (right-deviated or left-deviated). Laterally deviated apertures capture scattered light that returns indirectly from the illuminated fundus and blocks the directly backscattered light from the confocal plane [9,10]. RM-SLO represents a modification and advancement of the indirect mode (so-called “dark-field” mode), characterized by a central stop and a full ring aperture (annular ring aperture, RA), because of the repeatability and the higher contrast of the images as consequence of its fully modified confocal aperture [8,11]. Retromode (RM) and dark-field (DF) imaging are both based on the principles of retro-illumination, where images are created from light transilluminating chorioretinal structures (retroillumination) rather than reflected off their inner surfaces (Figure 1) [12]. Principles of RM imaging suggest that it may enable the detection of pigmented choroidal lesions, due to the blocking of backscattering illumination. However, this specific detection ability has not yet been evaluated.

The purpose of this study was to analyze the morphological features of choroidal nevi using RM-SLO imaging and to compare these findings with those obtained using other imaging modalities (multicolor fundus, fundus autofluorescence, infrared, dark-field imaging and optical coherence tomography scan), acquired with the cSLO Nidek Mirante.

## 2. Materials and Methods

We performed an observational, single-center study which conformed to the current version of the Declaration of Helsinki and to the Health Insurance Portability and Accountability Act regulations, as well as all national legal and regulatory requirements. All participants were informed as to the aim of the study and signed informed consent for the use of the data obtained during the ophthalmic examination.

We included patients with choroidal nevi who were referred to the Department of Ophthalmology, Istituto Clinico Città Studi of Milan, between September 2022 and November 2022. The diagnosis of choroidal nevus was obtained by a combination of clinical features and multimodal imaging characteristics. All patients underwent a complete slit-lamp and fundoscopic examination. Multimodal imaging was performed using a confocal scanning laser ophthalmoscope platform from Nidek Mirante (cSLO, Nidek Co., Ltd., Gamagori, Japan). Clinical data were collected at initial examination, including patient age, gender, race, and ethnicity. If an eye had more than one nevus, then the largest nevus was included in the analysis. Exclusion criteria were patients with poor-quality images, eyes with lesions beyond the equator and in the far periphery that were only partially visible in any imaging modality, myopia more than 8 diopters, and hyperopia more than 4 diopters.

### 2.1. Image Acquisition and Grading

Retromode (RM), dark-field (DF), multicolor fundus (mCF), fundus autofluorescence (FAF), infrared reflectance (IR) and spectral-domain optical coherence tomography (OCT) were performed for each choroidal nevus on the same day with cSLO Nidek Mirante. The FlexTrack algorithm available on the device was used to correct image distortion due to unstable fixation and to enhance averaging quality. Imaging was performed by a certified technician (CA) in all cases. All Nidek Mirante images used in the study had a field of view of 45 degree and a resolution of 2048 × 2048 pixels for RM, DF, mCF and IR images and 1536 × 1536 pixels for FAF images.

**Figure 2:**
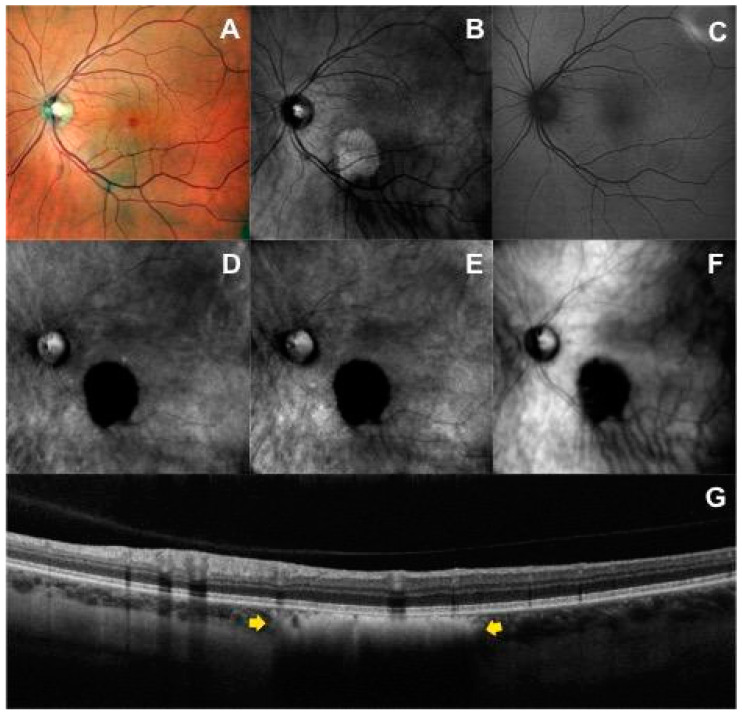
Multimodal imaging of a patient with choroidal nevus in the left eye: (**A**) Multicolor fundus image shows a lightly and heterogeneous pigmented area with barely visible lesion borders localized inferior to the fovea. (**B**) Infrared image shows a hyperreflective mass. (**C**) On the green fundus autofluorescence image, the nevus is not visualized. Left-deviated (**D**) and right-deviated (**E**) retromode images show the characteristic hypo-retro-reflective pattern within the choroidal nevus characterized by a dense dark shadow with sharp margins, shared also with dark-field mode (**F**). (**G**) The horizontal optical coherence tomography scan passing through the lesion identifies the choroidal nevus (yellow arrows) as a flat hyperreflective lesion with posterior shadowing, an intact overlying retinal pigment epithelium, and intact outer retinal layers.

**Figure 3:**
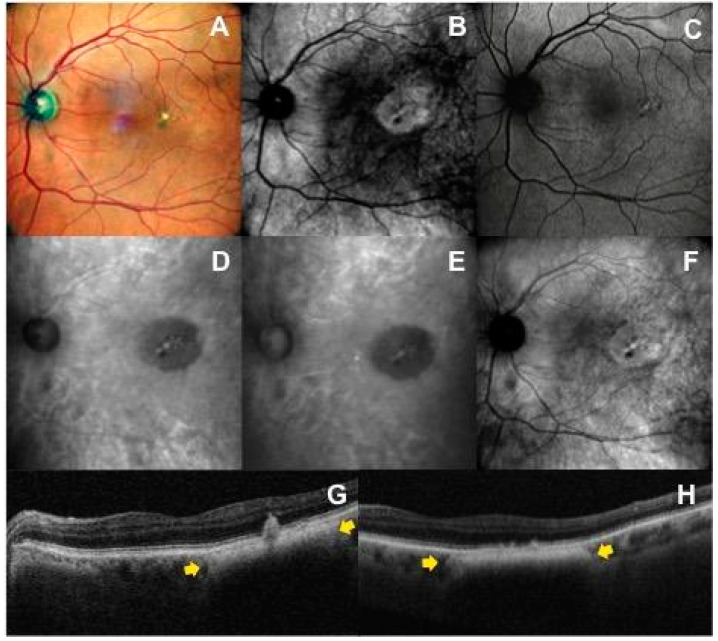
Multimodal imaging features in a patient with juxtafoveal choroidal nevus with overlying drusen in the left eye: (**A**) The nevus is not visible in the multicolor fundus image, except for the presence of multiple yellow drusen-like deposits and pigmentary rearrangement overlying the mass. (**B**) In the infrared reflectance image, the nevus is well defined as a round hyperreflective area. (**C**) Fundus autofluorescence imaging cannot image the tumor itself, but shows secondary overlying RPE and retinal changes. Left-deviated (**D**) and right-deviated (**E**) retromode images highlight a hypo-retro-reflective mass with a less intense gray shadow and overlying flecks. (**F**) On dark-field imaging, the choroidal nevus shows an iso-retro-reflective pattern with flecks. (**G**,**H**) The corresponding horizontal OCT scans show the choroidal nevus (yellow arrows) and the overlying drusen-like deposits accompanied by changes at the level of the retinal pigment epithelium (e.g., RPE migration, RPE clumping).

As previously discussed, RM-SLO consists of a modified version of the DF mode where only a small part of the annular aperture is used with a deviation to the right (DR) or to the left (DL) sides. Three different images per nevus were consecutively obtained via three different apertures: retromode (RM) with the DR and DL aperture and dark-field (DF) with RA aperture. These images were graded as hyper-retro-reflective if the reflectivity was brighter than the background, iso-retro-reflective if it was the same brightness as the background, and hypo-retro-reflective if the reflectivity was less bright than the background. Hypo-retro-reflective nevi were divided into two groups: gray and dark lesions according to the intensity of the shadow. Figure 2 and Figure 3 show the features of imaging retro-reflectivity. In addition, the visualization of the lesion border was scored using a grading scale between 0 and 2. “Grade 0” was given when the lesion margin was not visible on the imaging modality, “Grade 1” indicated that the margin of the lesion was barely visible or blurred, and “Grade 2” indicated that the margin was clearly visible and sharp. The same grading scale was also used for other imaging modalities. Finally, the presence of flecks, defined as hyper-retro-reflective spots within the tumor margin, was recorded.

Multicolor fundus images were generated using the built-in software combining three different laser wavelengths: blue (488 nm), green (532 nm) and red (670 nm), coupled with a specifically dedicated detector for each wave-length. The location of choroidal nevi (only assessed if located in the postequatorial fundus), the degree of pigmentation, the visualization of the lesion border, the presence of orange pigment (lipofuscin) and the presence of drusen or retinal pigment epithelium defects were noted on mCF. Infrared reflectance images were acquired using a 790 nm diode laser. IR features recorded included visibility of the lesion, appearance of the lesion (hyporeflective, isoreflective or hyperreflective), according to the description by Vallabh NA et al. [13], presence of flecks (defined as hyperreflective spots within tumor margin), presence of hyporeflective halo (defined as dark circumferential ring around nevus) and visualization of the lesion border. Fundus autofluorescence was obtained through a green light excitation wavelength of 532 nm, chosen to reduce the potential interference from macular pigment [14] in macular or juxtamacular nevi. FAF features recorded included visibility of the lesion and the presence of hyper-autofluorescent flecks.

All patients underwent OCT to detect the presence of subretinal fluid (SRF) within 3 mm from tumor margin, intraretinal fluid, drusen and RPE defects overlying the tumor and the surface configuration of the tumor (flat, dome-shaped, lumpy bumpy, excavated).

Two ophthalmologist consultants (CA and FC) independently evaluated each image by identifying and recording the morphological features associated with each choroidal nevus. In cases of discrepancy, the final decision was adjudicated by a third observer (PC).

### 2.2. Statistical Analysis

Statistical analysis was performed with SPSS for Macintosh software (version 25.0, IBM SPSS Inc., Chicago, IL, USA) and GraphPad Prism version 7.0 for Macintosh (GraphPad software, San Diego, CA, USA). All quantitative variables were presented as mean and standard deviation in the results and tables. Categorical data were compared using Pearson’s chi-squared or Fisher’s exact test. We determined sensitivity and specificity of Nidek Mirante Retromode Imaging in detecting drusen using OCT as the golden standard. The Kendall tau-b test was used to measure the strength of association between measures. In all cases, a *p* < 0.05 was considered statistically significant.

## 3. Results

Forty-one eyes (41 patients) with a clinical diagnosis of choroidal nevus were included in the study. Twenty-three (56.1%) were female, 18 (43.9%) were male, and the mean age was 74.73 ± 9.88 years. Forty patients (97.6%) were non-Hispanic white, while one patient was Hispanic white. All the patients underwent RM (DR and DL aperture), DF (RA aperture), mCF, FAF, IR and OCT imaging. However, OCT was not correctly performed for one nevus, and RM-DR was not available for another nevus.

### 3.1. Retromode and Dark-Field Imaging

In 100% of available images, RM (DR and DL aperture) and DF (RA mode) were able to detect choroidal nevi. In RM DR aperture and DL aperture, 97.6% and 100% of nevi appeared as hypo-retro-reflective lesions, respectively. Choroidal nevi were characterized by a dense dark shadow (Figure 2D,E) on RM DR and DL aperture in 37 of 41 cases (90.2%) and by a less intense gray shadow (Figure 3D,E) in the remaining cases. In RA mode, 87.8% of nevi were hypo-retro-reflective, with 27 of 41 (65.9%) appearing as a dark shadow and 9 of 41 (22.0%) as a gray shadow. In contrast to DL and DR-mode, in RA mode, 7.3% (3/41) of lesions were iso-retro-reflective (Figure 3F) and 4.9% (2/41) were hyper-retro-reflective. The greater variability in the level of retro-reflectivity on DF (RA mode) compared to RM (DR and DL) was statistically significant (*p* = 0.015).

In 100% of available images, RM and DF were able to delineate the border of the lesion. In 82.9% of eyes, margins were clearly visible with sharp edges in both RM-DL and RM-DR images; this percentage was slightly lower in DF images (70.7%). Flecks were detected in 14.6% of nevi on RM-DR, in 19.5% on RM-DL and in 36.6% on DF images. The RM (DR and DL mode) and DF (RA mode) characteristics of the nevi lesions are reported in Table 1.

### 3.2. Correlation between Imaging Modalities

#### 3.2.1. Spectral-Domain Optical Coherence Tomography Imaging

On OCT, drusen overlying the nevus were noted in 20 of 41 patients (48.8%) and none of the lesions demonstrated subretinal fluid. Nearly all nevi (40 of 41) had an intact overlying inner retina, while one case (2.4%) demonstrated overlying intraretinal cystoid changes. On B-scan images, seven choroidal nevi (17.1%) showed a dome-shaped configuration with overlying elevation of the RPE, while the other thirty-three cases (73.2%) were flat. There was no correlation between elevation of the RPE on OCT and level of retro-reflectivity (RM-DR *p* = 0.54, RM-DL *p* = 0.45, DF *p* = 0.76), visualization of border (RM-DR *p* = 0.72, RM-DL *p* = 0.65, DF *p* = 0.06) and presence of flecks (RM-DR *p* = 0.71, RM-DL *p* = 0.43, DF *p* = 0.22) on RM and DF images. Statistical analysis confirmed the relationship between presence of drusen on OCT images and flecks in RM and DF images with high specificity (100% RM-DR, τ = 0.45 *p* = 0.006; 100% RM-DL, τ = 0.46, *p* = 0.002; 90% DF, τ = 0.58, *p* < 0.0001) but a lower sensitivity (32.6% RM-DR, 40% RM-DL, 65% DF): as not all drusen deposits highlighted in OCT scans were consistently detected in RM images (20/41 on OCT, 6/40 on DR-RM and 8/41 on DL-RM).

#### 3.2.2. Multicolor Fundus Imaging

Most nevi were located along the vascular arcades (23 of 41, 56.1%); 7 of 41 (17.2%) were juxtapapillary with tumor margin within 3 mm to the optic disk, 5 of 41 (12.2%) were located at the posterior pole, 4 of 41 (9.8%) were in the mid periphery between the vascular arcades and the equator and 2 of 41 (4.9%) were in the macula. Chi-squared analysis showed no correlation between RM and DF appearance in relation to choroidal nevus location. By using mCF imaging, 17 nevi (41.5%) were classified as well-pigmented lesions; they appeared as gray-brown colored areas and ranged from homogeneous to more heterogeneous lesions with secondary changes overlying the nevus, such as drusen and pigment mottling. 13 of 41 (31.7%) were partially or lightly pigmented and 11 of 41 (26.8%) were not visible at all. None of the studied eyes showed orange pigment overlying the nevus on mCF images, which was also confirmed on FAF. There was no correlation between nevus appearance on RM and pigmentation degree on mCF images (*p* = 0.235 DR-RM, *p* = 0.538 DL-RM).

#### 3.2.3. Infrared Reflectance Imaging

On IR, most choroidal nevi appeared as hyperreflective lesions (56.1%), 15 of 41 (36.6%) were isoreflective, 1 of 41 (2.4%) was hyporeflective and 2 of 41 (4.9%) were not visible. A hyporeflective halo around the nevus was detected in 24.4% and flecks within tumor margin were detected in 22% of nevi. No correlation was found between IR reflectivity and RM retro-reflectivity (*p* = 0.961 DR-RM, *p* = 0.903 DL-RM).

#### 3.2.4. Fundus Autofluorescence Imaging

On FAF, a normal pattern of background fundus autofluorescence with no corresponding areas of hyperautofluorescence or hypoautofluorescence over the nevi was found. Eleven patients (26.8%), however, had faint localized hyperautofluorescent areas that corresponded to small drusen, drusenoid pigment epithelium detachments or RPE irregularities.

#### 3.2.5. Visualization of Lesion Border

**Figure 4:**
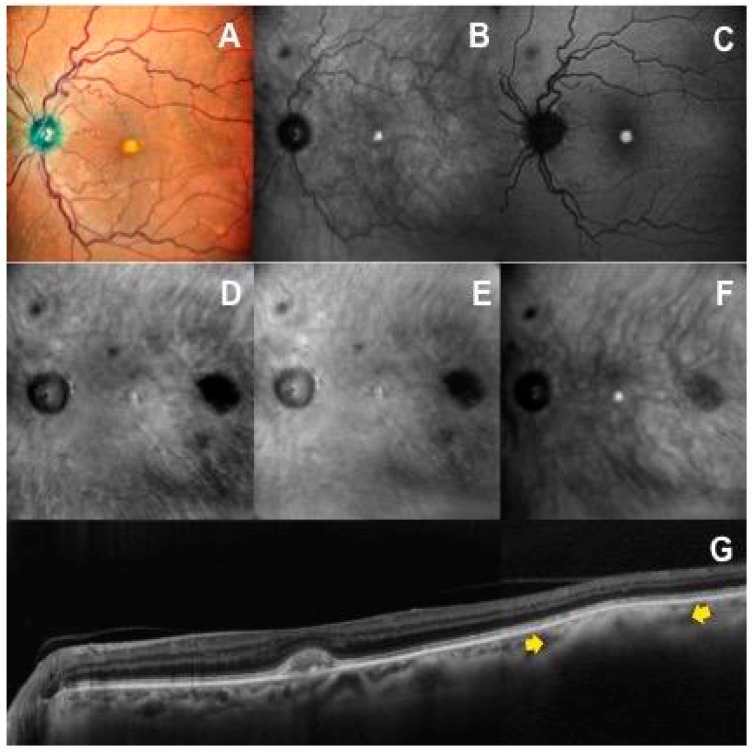
A case with a small choroidal nevus in the posterior pole of the left eye not visible on multicolor fundus (**A**), infrared reflectance (**B**) and autofluorescence (**C**) images. The choroidal nevus is clearly detectable on retromode images (**D**,**E**) as a dark shadow temporal to the macula, on dark-field image (**F**) as a gray shadow, and on OCT scan (**G**) as an hyperreflective lesion with posterior shadowing (yellow arrows).

The mCF image did not allow for visualization of the choroidal nevus margins in 13 (31.7%) eyes. Only 15 of 41 (36.5%) eyes had sharp and well-defined margins, compared to 100% of detectable choroidal nevus lesion and the highest rate of sharp lesion borders (82.9% on DR and DL-RM, 70% on DF) on the available RM and DF images. On IR retinography, four (9.8%) nevi were not visible, and 27 of 41 (65.9%) showed clearly defined margins (Table 2). In two cases, the choroidal nevi incidentally detected on OCT scan also appeared on RM and DF imaging but not on mCF, IR and FAF imaging (Figure 4).

## 4. Discussion

In previous research, RM imaging has been used to evaluate pathologic changes in several retinal and choroidal diseases, including RPE alterations in central serous chorioretinopathy, macular retinoschisis in myopic maculopathy, diabetic macular edema, abnormalities in polypoidal choroidal vasculopathy, hydroxychloroquine retinopathy, age-related macular degeneration and drusen, sites of subthreshold laser scars and abnormalities in a variety of chorioretinal dystrophies [15,16,17,18,19,20,21]. These prior studies indicated that RM imaging could be useful in studying deep retinal pathologies, RPE and choroid changes due to the use of infrared light, which allows for deeper penetration than shorter wavelengths [7,8,10].

Choroidal nevi are benign tumors arising from melanocytic cells of the choroid that are sometimes associated with overlying RPE alterations and drusen [22]. The present study documented pathologic findings on RM imaging and evaluated its diagnostic validity in choroidal nevi diagnosis comparing different imaging techniques through the platform of the confocal scanning laser ophthalmoscope Nidek Mirante (cSLO, Nidek Co., Ltd., Gamagori, Japan). We found that RM-SLO is a reliable technique for noninvasive evaluation of choroidal nevi, providing several important findings in these patients.

In our case series, an RM-SLO hypo-retro-reflective pattern was a characteristic finding that was observed in all choroidal nevi (100% of available images). Choroidal nevi appeared as well-defined, round, dark (about 90% of cases) or gray lesions on RM-DR and RM-DL. Interestingly, even choroidal nevi not visible on multicolor fundus, infrared and fundus autofluorescence images were clearly identified on RM-SLO, where they demonstrated the typical hypo-retro-reflective pattern (Figure 4). The mechanism underlying the retromode display of nevi seems to be a blocking effect of infrared light by pigmented cells and melanocytes that compose the nevus, located at varying depths within the choroid. We know that the absorption spectrum of melanin is maximum for visible light (low wavelength), while infrared light (high wavelength) has the least absorption and maximum reflectance [23]. Thus, the light backscattered from the inner sclera is blocked by nevi containing higher amounts of melanin and penetrates the whole RPE/choroid complex in the surrounding normal retina to return back to the instrument detector. We speculate that nevi containing increased melanocytes with normal melanin content could have been detected as lighter shadow area (gray shadow), but instead retromode nevus appearance was not found to be statistically related to the pigmentation degree on mCF images or the reflectivity degree on IR, as well as independent to location and surface configuration of choroidal nevus.

On DF imaging, which shares the same basic principle of retroillumination as RM [9,10,12], choroidal nevi showed greater variability in appearance upon imaging. On RM-SLO, all lesions had a characteristic hypo-retro-reflective pattern, while on DF-SLO, some choroidal nevi were hyper- or iso-retro-reflective.

Previous studies have already discussed the ability of RM-SLO to detect various types of drusen, to depict their three-dimensional shape and to appreciate their geographic distribution in age-related macular degeneration. In some studies, RM imaging was even able to detect significantly more drusen than conventional color fundus photography [24,25,26]. In the current series, most nevi had a homogeneous dark internal aspect on RM imaging, although in some cases multiple small, generally hyper-retro-reflective dots overlying the nevus were noted (Figure 3). Comparing RM-SLO with OCT, we found that these flecks corresponded to drusen, subretinal drusenoid deposits or RPE defects with high specificity. However, RM-SLO did not highlight all cases of drusen, demonstrating lower sensitivity in detecting this feature compared to OCT. We speculate that this may be secondary to nevus pigment interfering in acquisition of RM imaging. Additionally, on DF-SLO, not all drusen deposits were consistently detected, but the rate was significantly higher compared with RM-SLO images. This could be because fewer choroidal nevi demonstrated a dense dark shadow pattern on DF imaging.

Among the imaging methods examined, RM-SLO and DF-SLO showed the highest sensitivity for the detection of choroidal nevi (100% on RM, 100% on DF versus 68% for mCF and 90.2% on IR), identifying even the smallest nevi in any location. The ability of DF-SLO to detect choroidal nevi with a high sensitivity has already been discussed in a previous study [27]. In that study, DF-SLO also showed a high repeatability in the linear measurements of nevi, which were described as dense or partially transparent shadow on DF-SLO, which is in accordance with our findings. IR imaging demonstrated the presence of choroidal nevi in 90.2% of cases, most commonly as hyperreflective areas, which is consistent with findings from prior series [13]. On green autofluorescence, the margins of the lesions were not visible, but this imaging modality was helpful in identifying retinal and RPE changes associated with choroidal nevus, as previously described [28,29].

It is important to note that the cSLO Nidek Mirante platform does not provide conventional color fundus photography obtained with a white flashing light, but rather a multicolor fundus image, generated using built-in software combining three different laser wavelengths (blue, green and red). In a previous study, it was found that the appearance of choroidal nevi on multicolor imaging might differ from the appearance on color fundus photography and on fundus examination. In particular, pseudocolor images demonstrate some significant differences in terms of pigmentation of retinal and choroidal lesions compared to conventional fundus photography [30,31]. To the best of our knowledge, there is no comparative study between choroidal nevi imaged with Nidek Mirante cSLO-mCF and conventional color fundus photography, and investigations in the future may be warranted.

Among all imaging modalities examined, RM-SLO was best able to delineate the margins of lesions, demonstrating the highest rate of sharpness and accuracy (82.9% on DR-RM and DL-RM, 70% on DF, 36.6% on mCF, 65.9% on IR of the available images showed clearly defined margins). This is an essential feature of imaging for oncologic diseases, in which accurate observation on follow-up is critical for assessing any progression in basal dimensions and/or thickness. Small choroidal melanomas can be difficult to differentiate from choroidal nevi even with multimodal imaging [3], making growth over short periods of observation critical for the diagnosis. Furthermore, the size of the nevus was recently proposed as a risk factor for malignant transformation of choroidal nevi into melanoma by Shields et al. in a retrospective chart review and follow-up of 2355 cases using multimodal imaging [4].

We must also note that RM-SLO is a noninvasive, simple and rapid imaging technique. The infrared light used in RM imaging allows for nonmydriatic examination. It is also advantageous in elderly patients with lens opacities, since light is minimally scattered in the presence of media opacities. Patients are overall comfortable during the examination because the infrared light is not particularly bright and there is no flash. Moreover, it may be timesaving when patients need to undergo multiple imaging modalities, because they can be performed nearly simultaneously using the Nidek Mirante imaging platform.

All these features, in addition to the excellent potential in identifying choroidal nevi even if poorly pigmented, suggest the effectiveness of RM-SLO as a possible screening test for choroidal nevi. Moreover, monitoring the progression of the hypo-retro-reflective areas on RM may enhance serial observation for progression.

The limitations of our study include the relatively small sample size of the study group, its retrospective nature, and the lack of comparison with traditional color fundus photography. Further studies including a larger number of cases, as well as comparison with other imaging modalities from other instruments, might corroborate these findings and highlight the utility of RM-SLO as a fast, reliable and non-invasive way to diagnose and monitor choroidal nevi.

## 5. Conclusions

To the best of our knowledge, this is the first report investigating the characteristics of choroidal nevi using retromode imaging. RM imaging is a fast, reliable, and non-invasive diagnostic modality, that has demonstrated high sensitivity in detecting choroidal nevi, even when poorly pigmented. RM-SLO also allows to clearly and accurately delineate the margins of the lesions and to identify some typical characteristics such as drusen. These findings suggest that RM-SLO could represent an innovative and effective diagnostic imaging modality in choroidal nevi screening and follow-up. 

## Figures and Tables

**Table 1:** Level of retro-reflectivity on different imaging modalities.

	Retromode-DR (DR Aperture)	Retromode-DL (DL Aperture)	Dark-Field (RA Aperture)
Level of retro-reflectivity			
Hypo-retro-reflective	40/41 (97.6%)	41/41 (100%)	36/41 (87.8%)
- Dark shadow	37/41 (90.2%)	37/41 (90.2%)	27/41 (65.9%)
- Gray shadow	3/41 (7.3%)	4/41 (9.8%)	9/41 (22.0%)
Iso-retro-reflective	0	0	3/41 (7.3%)
Hyper-retro-reflective	0	0	2/41 (4.9%)
Not available	1/41 (2.4%)	0	0

**Table 2:** The distribution of visibility of choroidal nevi and borders using multimodal imaging.

	Multicolor	Infrared	Retromode-DR (DR Aperture)	Retromode-DL (DL Aperture)	Dark-Field (RA Aperture)
Visualization of nevus					
- Not available	0	0	1/41 (2.4%)	0	0
- Not visible	13/41 (31.7%)	4/41 (9.8%)	0	0	0
- Visible	28/41 (68.3%)	37/41 (90.2%)	40/41 (97.6%)	41/41 (100%)	41/41 (100%)
Visualization of border					
- Barely visible	13/41 (31.7%)	10/41 (24.4%)	6/41 (14.6%)	7/41 (17.1%)	12/41 (29.3%)
- Clearly visible	15/41 (36.6%)	27/41 (65.9%)	34/41 (82.9%)	34/41 (82.9%)	29/41 (70.7%)

## Data Availability

The data used to support the findings of this study are available from the corresponding author upon reasonable request.

## References

[1] Sumich P., Mitchell P. (1998). Choroidal nevi in a white population: The Blue Mountains Eye Study. Arch. Ophthalmol..

[2] Chien J.L., Sioufi K. (2017). Choroidal nevus: A review of prevalence, features, genetics, risks, and outcomes. Curr. Opin. Ophthalmol..

[3] Singh A.D., Kalyani P. (2005). Estimating the risk of malignant transformation of a choroidal nevus. Ophthalmology.

[4] Shields C.L., Dalvin L.A. (2019). Choroidal nevus imaging features in 3,806 cases and risk factors for transformation into melanoma in 2,355 cases: The 2020 Taylor R. Smith and Victor T. Curtin Lecture. Retina.

[5] Geiger F., Said S. (2022). Assessing Choroidal Nevi, Melanomas and Indeterminate Melanocytic Lesions Using Multimodal Imaging-A Retrospective Chart Review. Curr. Oncol..

[6] Li X., Wang L. (2021). Application of Multimodal and Molecular Imaging Techniques in the Detection of Choroidal Melanomas. Front. Oncol..

[7] Elsner A.E., Burns S.A. (1996). Infrared imaging of sub-retinal structures in the human ocular fundus. Vis. Res..

[8] Geeraets W.J., Williams R.C. (1960). The loss of light energy in retina and choroid. Arch. Ophthalmol..

[9] Lee W.J., Lee B.R. (2014). Retromode imaging: Review and perspectives. Saudi J. Ophthalmol..

[10] Mainster M.A., Desmettre T. (2022). Scanning laser ophthalmoscopy retroillumination: Applications and illusions. Int. J. Retin. Vitr..

[11] Elsner A.E., Burns S.A. (1992). Reflectometry with a scanning laser ophthalmoscope. Appl. Opt..

[12] Woon W.H., Fitzke F.W. (1992). Confocal imaging of the fundus using a scanning laser ophthalmoscope. Br. J. Ophthalmol..

[13] Vallabh N.A., Sahni J.N. (2016). Near-infrared reflectance and autofluorescence imaging characteristics of choroidal nevi. Eye.

[14] Schmitz-Valckenberg S., Pfau M. (2021). Fundus autofluorescence imaging. Prog. Retin. Eye Res..

[15] Vujosevic S., Pucci P. (2014). Extent of diabetic macular edema by scanning laser ophthalmoscope in the retromode and its functional correlations. Retina.

[16] Su Y., Zhang X. (2014). The noninvasive retro-mode imaging of confocal scanning laser ophthalmoscopy in myopic maculopathy: A prospective observational study. Eye.

[17] Ohkoshi K., Tsuiki E. (2010). Visualization of subthreshold micropulse diode laser photocoagulation by scanning laser ophthalmoscopy in the retro mode. Am. J. Ophthalmol..

[18] Giansanti F., Mercuri S. (2022). Scanning Laser Ophthalmoscopy Retromode Imaging Compared to Fundus Autofluorescence in Detecting Outer Retinal Features in Central Serous Chorioretinopathy. Diagnostics.

[19] Pilotto E., Sportiello P. (2013). Confocal scanning laser ophthalmoscope in the retromode imaging modality in exudative age-related macular degeneration. Graefes Arch. Clin. Exp. Ophthalmol..

[20] Zeng R., Zhang X. (2013). The noninvasive retro-mode imaging modality of confocal scanning laser ophthalmoscopy in polypoidal choroidal vasculopathy: A preliminary application. PLoS ONE.

[21] Ahn S.J., Lee S.U. (2018). Evaluation of Retromode Imaging for Use in Hydroxychloroquine Retinopathy. Am. J. Ophthalmol..

[22] Shields C.L., Furuta M. (2008). Clinical spectrum of choroidal nevi based on age at presentation in 3422 consecutive eyes. Ophthalmology.

[23] Song W., Zhang L. (2017). Wavelength-dependent optical properties of melanosomes in retinal pigmented epithelium and their changes with melanin bleaching: A numerical study. Biomed. Opt. Express.

[24] Acton J.H., Cubbidge R.P. (2011). Drusen detection in retro-mode imaging by a scanning laser ophthalmoscope. Acta Ophthalmol..

[25] Corradetti G., Corvi F. (2021). Subretinal Drusenoid Deposits Revealed by Color SLO and Retro-Mode Imaging. Ophthalmology.

[26] Monteduro D., Cozzi M. (2019). Topographic detection and characterization of drusen and subretinal drusenoid deposits: Contribution of Retro mode modality in a multimodal imaging approach. Investig. Ophthalmol. Vis. Sci..

[27] Kulikov A.N., Maltsev D.S. (2019). Characterization of choroidal nevi with dark-field infrared scanning laser ophthalmoscopy. Ophthalmol. Retin..

[28] Bindewald-Wittich A., Holz F.G. (2022). Fundus Autofluorescence Imaging in Patients with Choroidal Melanoma. Cancers.

[29] Lavinsky D., Belfort R.N. (2007). Fundus autofluorescence of choroidal nevus and melanoma. Br. J. Ophthalmol..

[30] Muftuoglu I.K., Gaber R. (2018). Comparison of conventional color fundus photography and multicolor imaging in choroidal or retinal lesions. Graefes Arch. Clin. Exp. Ophthalmol..

[31] Venkatesh R., Pereira A. (2020). Variability in Imaging Findings in Choroidal Nevus Using Multicolor Imaging Vis-à-vis Color Fundus Photography. J. Curr. Ophthalmol..

